# *Vibrio vulnificus* necrotizing fasciitis with sepsis presenting with pain in the lower legs in winter: a case report

**DOI:** 10.1186/s12879-022-07655-1

**Published:** 2022-08-04

**Authors:** Weihua Di, Jing Cui, Hui Yu, Xiao Cui, Huanlan Sa, Zhong Fu, Bingjin Fu, Guofeng Guan, Rui Du, Cuijie Shao, Yong Gao

**Affiliations:** 1 Department of Pain, Binzhou Medical University Hospital, Binzhou, 256603 China; 2 Department of Rehabilitation Medicine, Binzhou Medical University Hospital, Binzhou, 256603 China; 3 Department of Intensive Care Unit, Binzhou Medical University Hospital, Binzhou, 256603 China; 4 Department of Foot and Ankle Surgery, Binzhou Medical University Hospital, Binzhou, 256603 China

**Keywords:** *Vibrio vulnificus*, *V. vulnificus* infection, Lower leg pain, Necrotizing fasciitis, Sepsis

## Abstract

**Background:**

*Vibrio vulnificus* infections develop rapidly and are associated with a high mortality rate. The rates of diagnosis and treatment are directly associated with mortality.

**Case presentation:**

We describe an unusual case of a 61-year-old male patient with chronic liver disease and diabetes who presented with a chief complaint of pain in both lower legs due to *V. vulnificus* infection in winter. Within 12 h of arrival, typical skin lesions appeared, and the patient rapidly developed primary sepsis. Despite prompt appropriate antibiotic and surgical treatment, the patient died 16 days after admission.

**Conclusion:**

Our case findings suggest that *V. vulnificus* infection should be suspected in patients with an unclear infection status experiencing pain of unknown origin in the lower legs, particularly in patients with liver disease or diabetes, immunocompromised status, and alcoholism.

## Background

*Vibrio vulnificus* is a gram-negative, halophilic, alkaliphilic marine bacterial pathogen commonly found in plankton and shellfish, especially oysters, which was first described in 1976. [1, 2]. *V. vulnificus* favorably grows in warm water and low-salinity areas in coastal regions; therefore, *V. vulnificus* infections show seasonal and regional patterns [3]. *V. vulnificus* infections are generally acquired by consuming contaminated seafood, particularly oysters. Wound infections can occur after skin lesions are exposed to contaminated seawater [4, 5].

In this report, we present an unusual case of necrotizing fasciitis with sepsis presenting with pain in the lower legs caused by *V. vulnificus* infection in winter in our hospital located in Binzhou, a coastal city in eastern China. The patient complained of pain in both lower legs without presenting with other prodromal symptoms upon admission. To the best of our knowledge, this clinical presentation is unusual and could alert clinicians to possible *V. vulnificus* infection in patients with an unclear infection status, who experience pain of unknown origin in the lower legs even before the appearance of typical skin lesions, especially if the patients have risk factors.

## Case presentation

A 61-year-old male patient was transferred to our hospital on November 29, 2020, at 23:30 pm, with complaints of pain in both lower legs for 16 h. He had a medical history of hepatitis B, hepatitis B-decompensated cirrhosis, and liver cancer, along with hypertension, diabetes mellitus, and tobacco use (2 packs of cigarettes per day). Moreover, he had a history of esophageal variceal ligation under endoscopic surgery 5 years earlier. He had consumed seafood (including turtle, hairtail, and shellfish) for lunch 3 days before admission.

The patient was in a normal state until the morning of the 3rd day after consuming seafood with his family when he developed pain in both lower legs on awakening. He could recall no traumatic events or any history of recreational marine exposure. He did not consider the pain as a concern and ingested 800 mg of ibuprofen and sprayed diclofenac sodium on his lower legs. He experienced worsening of pain and arrived at our hospital at night. Color ultrasound examination of the legs showed normal findings.

Initial physical examination revealed the following: clear consciousness; body temperature, 36.5 °C; respiration rate, 17 breaths per minute; blood pressure, 92/50 mmHg; and heart rate, 68 beats per minute. Reddish patches were observed on the right ankle.

The initial laboratory investigations revealed the following: white blood cell count, 6.1 × 10^9^/L (94.3% neutrophils, 2.9% lymphocytes, and 2.8% monocytes); platelet count, 35 × 10^9^/L; hematocrit, 28%; serum glucose, 8.73 mmol/L; alanine aminotransferase, 22.84 U/L; ceramic oxaloacetic transaminase, 34.3 U/L; total bilirubin, 32.7 µmol/L (direct bilirubin, 9.1 µmol/L; indirect bilirubin, 23.6 µmol/L), serum γ-glutamyltransferase, 101.4 µmol/L; prothrombin time, 18.8 s; partial thromboplastin time, 43.5 s; internationally standardized ratio, 1.64; d-dimer, 3.07 mg/L; serum urea nitrogen, 14.88 mmol/L; myoglobin, 672.2 ng/mL; creatine kinase, 210.2 U/L; C-reactive protein, 223.90 mg/L; blood sodium, 124 mmol/L; blood chlorine, 93.90 mmol/L; and serum potassium, 5.52 mmol/L. Hemoglobin, hematocrit, creatinine, and routine urine test results were normal.

Blood test results indicated thrombocytopenia, unclear infection status, coagulation dysfunction, hypoalbuminemia, electrolyte disturbance, and renal dysfunction. Blood culture was performed, and empirical antibiotic therapy was initiated. We used levofloxacin (600 mg, qd) intravenously.

**Fig. 1:**
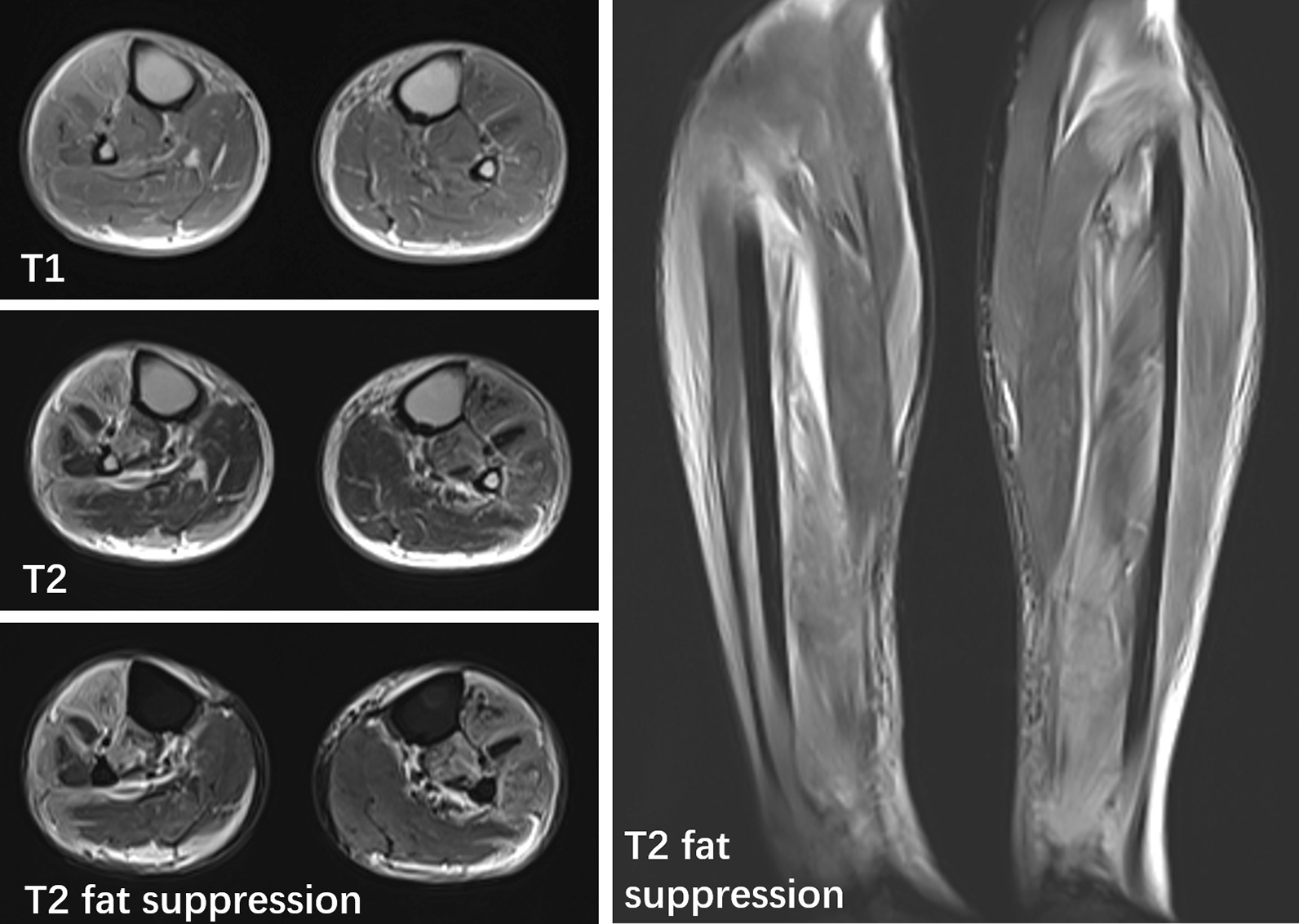
MRI showing extensive swelling in the soft tissue of the lower legs. *MRI* magnetic resonance imaging

**Fig. 2:**
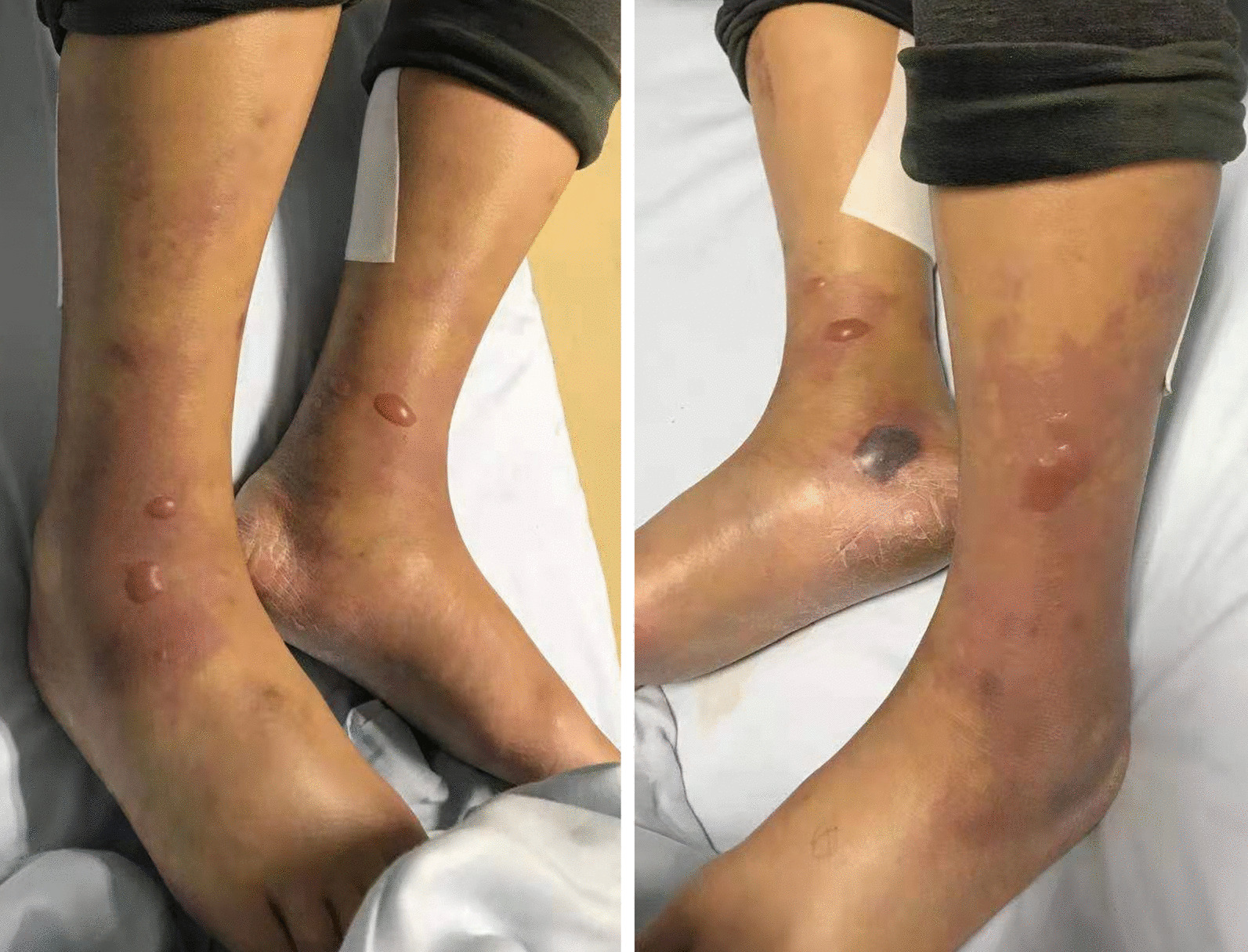
Twelve hours after admission: Bullae and erythematous plaque in both lower legs

**Fig. 3:**
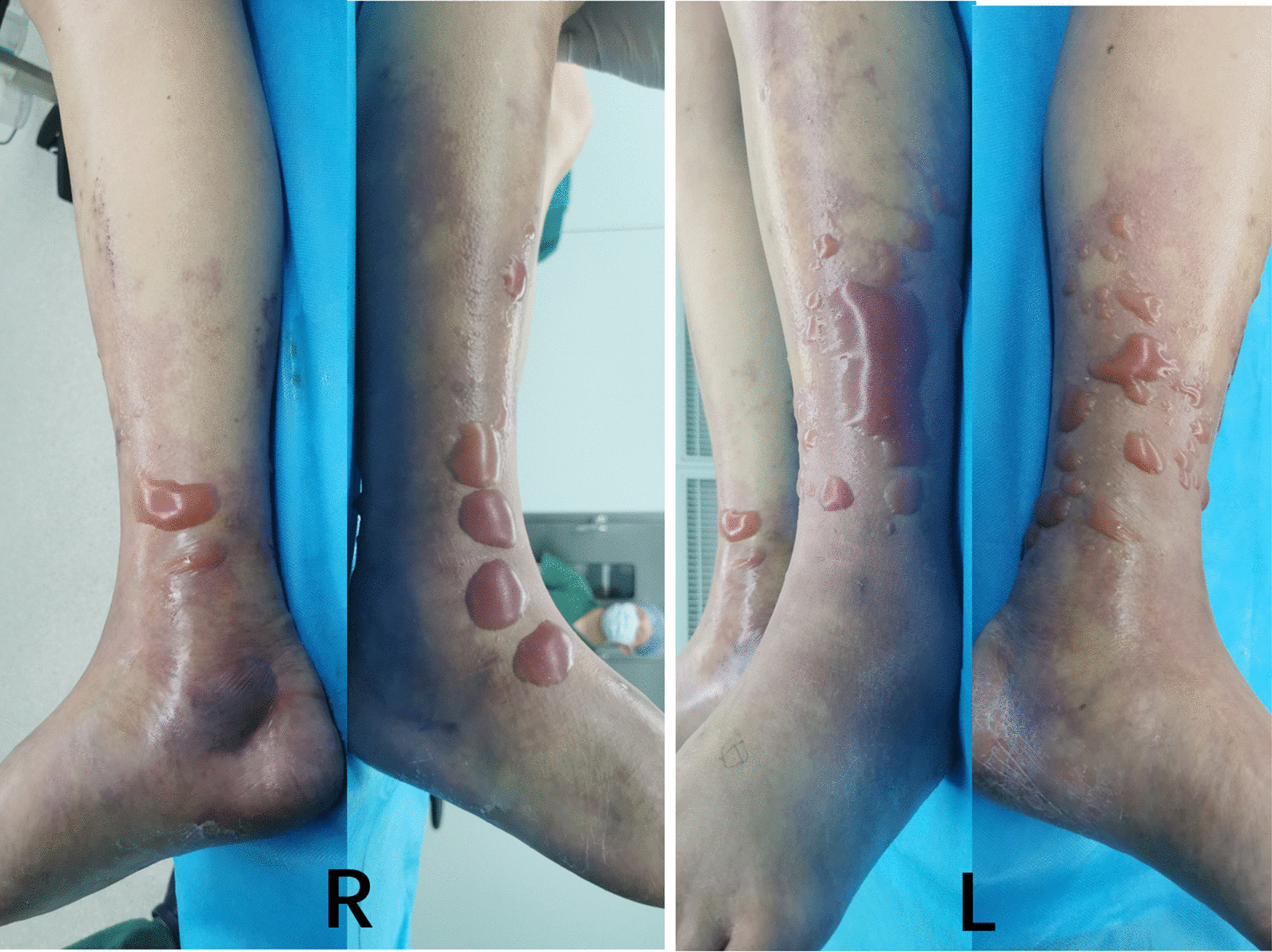
Fifteen hours after admission: Progression of the skin lesions in both legs

**Fig. 4:**
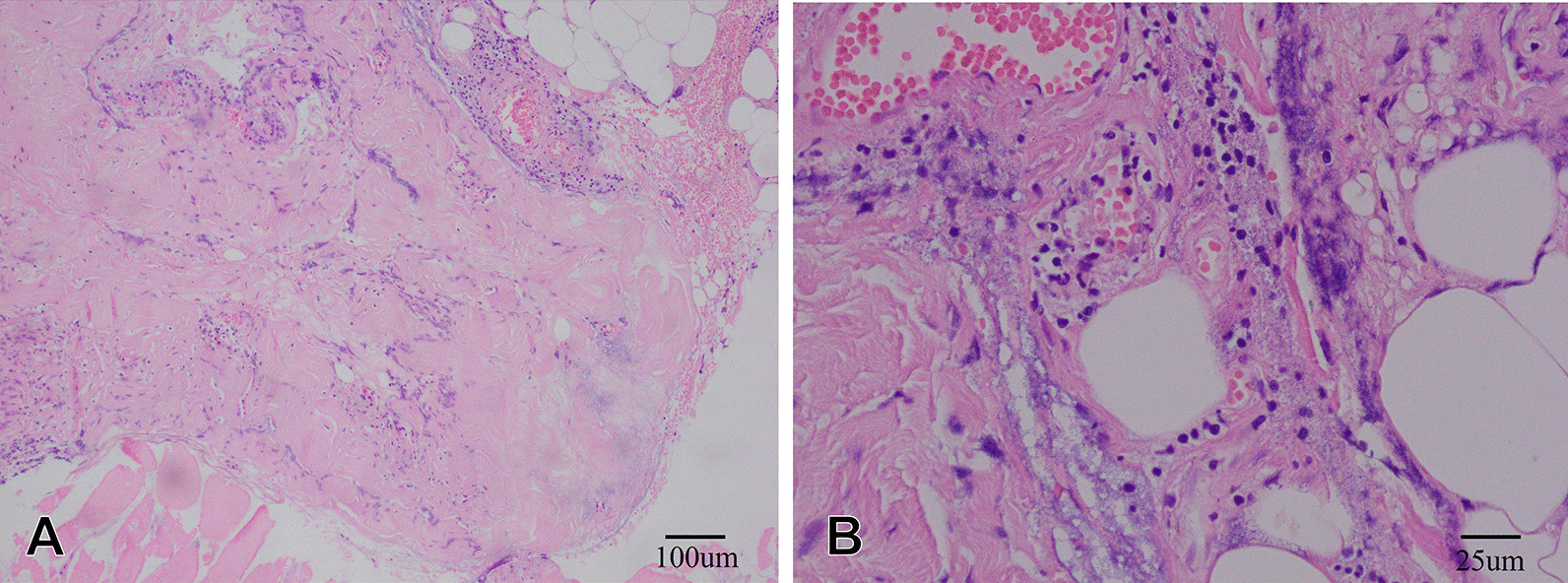
Histopathological findings: Swollen muscle fibers, dissolved necrotic sarcoplasm, high bacterial concentration in the muscle and surrounding adipose tissue, extensive infiltration of acute and chronic inflammatory cells, and dilated blood vessels with congested vasculitis. **A** Hematoxylin and eosin (H&E) stain, ×100. **B** H&E stain, ×400

Within 12 h of arriving to the hospital, the patient experienced swelling and severe pain. Creatine kinase increased to 14,141 U/L, and his white blood cell count decreased to 1.5 × 10^9^/L. Color ultrasound and magnetic resonance imaging of the legs revealed extensive swelling in the soft tissue of the lower legs, suggesting cellulitis and edema (Fig. 1). Several tense bullae, erythematous plaques, color changes in both lower legs, and hemorrhagic bullae in the right ankle were observed (Fig. 2). Subsequently, the patient continued to rapidly develop skin lesions, bullae, and vesicles even after 3 h (Fig. 3), and he further complained of decreased strength and paresthesia of the legs. Urgent surgical debridement and decompression were performed. During the operation, fishy odor was detected in the calf muscles and plantar compartments. Some of the muscle and fascia tissues were necrotic, and light-yellow fluid exudate was noted; no abscess was formed. Biopsy of the muscle and fascia tissue of both lower legs revealed swollen muscle fibers, dissolved and necrotic sarcoplasm, considerable bacterial concentration in the muscle and the surrounding adipose tissue, infiltration of extensive acute and chronic inflammatory cells, and dilated blood vessels congested with vasculitis (Fig. 4). The images were collected by cellSens image acquisition system of OLYBUS microscope BX51. The resolution of images is 1920 × 1440.

Postoperatively, the patient’s condition deteriorated rapidly, with the development of multiple organ dysfunction syndrome (circulation, disseminated intravascular coagulation, respiratory, liver, and kidney), and he was immediately admitted to the intensive care unit. Intravenous therapy with imipenem/cilastatin sodium (2 g, q8h), cefoperazone sodium/sulbactam sodium (3 g, q6h), and levofloxacin was initiated. Four days after admission, bacterial cultures from the blood, bullae fluid, and tissues (matrix-assisted laser desorption/ionization–time of flight mass spectrometry) were all positive for *V. vulnificus*. Susceptibility was determined with Vitek2 Compact according to the recommendations of Clinical Laboratory Standards Institute, M45, third edition. The cultured microbes revealed sensitivity to all tested antibiotics, including piperacillin, ceftazidime, cefoperazone, cefepime, imipenem, meropenem, ciprofloxacin, levofloxacin, doxycycline, and tigecycline.

**Fig. 5:**
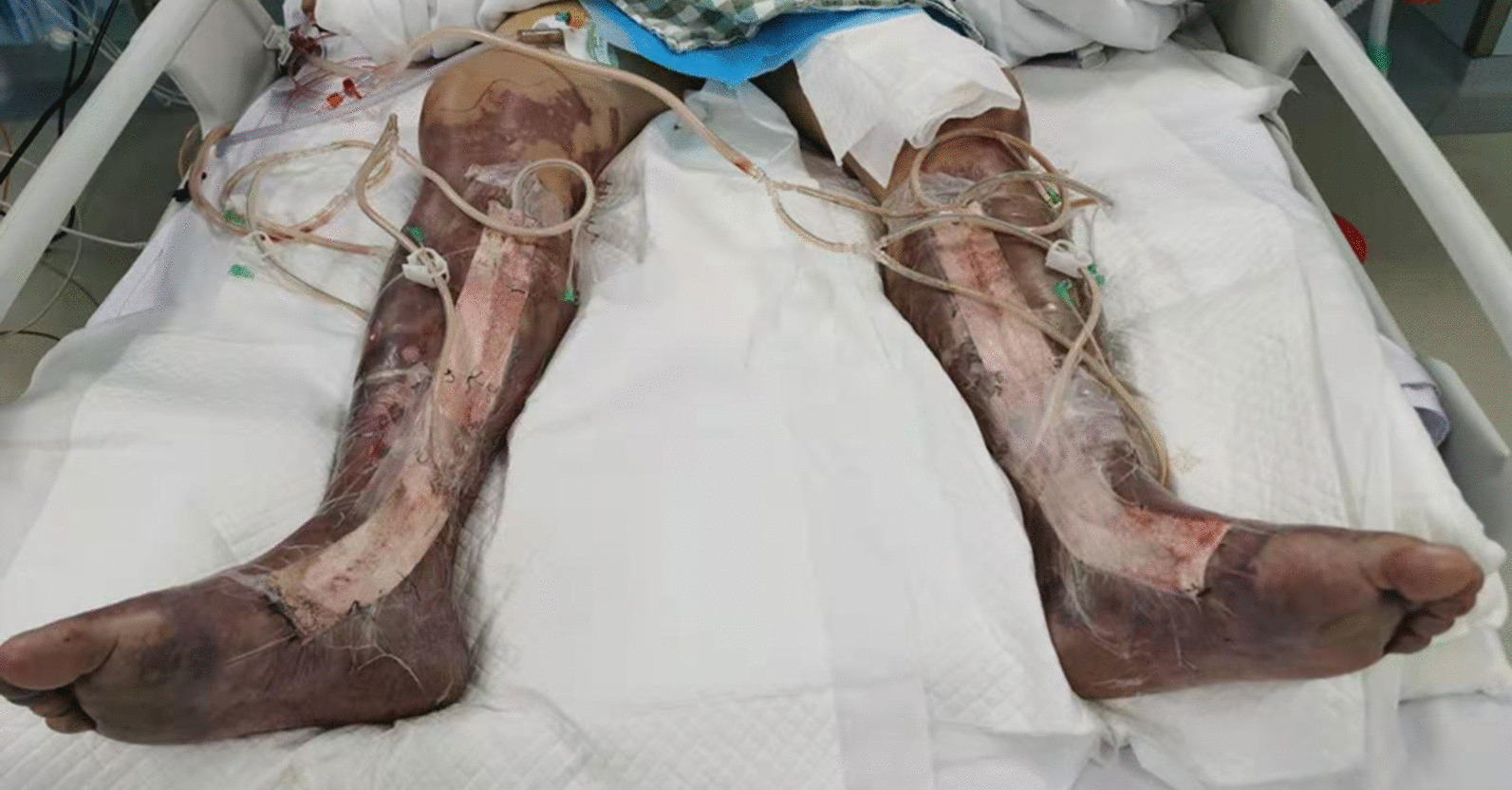
Day 4 after admission: progression of tissue necrosis to the thigh

Despite prompt appropriate antibiotic and surgical treatment, the skin lesions spread to the right thigh on the 2nd day after surgery (Fig. 5). Amputation was required; however, considering the progressively deteriorating condition and his previously verbalized wishes, his family refused amputation. Despite vigorous treatment, the patient died 16 days after admission. Postmortem examination was not performed.

## Discussion and conclusions

*Vibrio vulnificus* multiplies in approximately 10 min at 20 °C, and its multiplication is controlled below 15 °C [6]. *V. vulnificus* infections are common in warm climates, but our case occurred in winter. Although it is relatively unusual, there have been previous reports of cases in cold climates [7]; thus, we should pay attention to this disease even in winter.

Previous reports have shown that patients with liver disease are more susceptible to *V. vulnificus* infection, particularly those with hepatic cirrhosis. This may be due to the fact that patients with chronic liver disease often have portal hypertension, which can cause shunting of the bacteria around reticuloendothelial cells in the liver [8]. Other risk factors include diabetes, immunocompromised status, and alcoholism [5, 9, 10]. Moreover, it is believed that approximately 1 million bacilli are required to cause infection through oral ingestion [11]. The patient in our case was 61 years old, had an underlying liver disease and diabetes, and was at a high risk of infection. In addition, the man had a history of esophageal varix, which most likely indicates that he had portal hypertension, and that he may have had vascular ulceration in the gastrointestinal tract, making *V. vulnificus* more likely to enter the bloodstream and cause bacteremia on consumption of contaminated seafood. *V. vulnificus* disseminates from the gastrointestinal tract to the bloodstream, leading to the rapid development of primary sepsis. This may explain why only our patient developed primary sepsis 3 days after consuming seafood, although all his family members consumed the same diet.

Infection with this pathogen can result in three major discernible manifestations: primary sepsis, wound infections, and gastrointestinal diseases [1, 12]. Primary sepsis usually manifests as an acute symptom of systemic infection; it occurs within 24–48 h of ingesting the microorganism and often begins with prodromal symptoms, including watery diarrhea, fever, chills, nausea, vomiting, and abdominal pain, followed by skin lesions [9]. Typical skin lesions include local or flaky erythema and ecchymosis, blood blisters with exudation, necrosis and cellulitis, necrotising fasciitis, and muscle inflammation [13]. The wound infections due to *V. vulnificus* can progress to necrotizing soft-tissue infections [14]. The contact history of patients with a rapid onset of cellulitis can alert clinicians to a differential diagnosis of soft-tissue infection with *V. vulnificus* (contact with seawater or raw seafood) or Aeromonas species (contact with fresh or brackish water, soil, or wood) [15]. In addition, sepsis progresses rapidly, and most patients experience shock with hypotension at the time of hospital arrival.

In the present case, the patient presented with pain in both lower legs as the chief complaint. We did not detect any other prodromal symptoms; therefore, diagnosis was challenging until the development of the characteristic bullae. Despite the inapparent or atypical presentation, there were some indicators of infection. His blood pressure was slightly low, and laboratory examination findings suggested infection; these may indicate septic shock. Reddish patches on the right ankle, elevated d-dimer, and coagulation dysfunction may indicate progression to disseminated intravascular coagulation. Moreover, elevated creatine kinase may signify rhabdomyolysis. We should obtain critical information quickly when treating patients with suspected limb infections, which can help identify patients with life-threatening limb infections at an early stage.

Antibiotic therapy and debridement are the most effective methods for the treatment of necrotizing myofascitis caused by *V. vulnificus* infection. A recent study suggested that for patients presenting with septic shock with a recent history of raw seafood consumption, treatment with doxycycline or ciprofloxacin for appropriate coverage of *V. vulnificus* and gram-negative resistant organisms should commence while awaiting microbiological cultures. Once a diagnosis of *V. vulnificus* septicemia is confirmed, treatment can be safely changed to ceftriaxone combined with doxycycline or ciprofloxacin [16]. Early debridement plays a vital role in improving the prognosis of patients with *V. vulnificus* infection [17]. In the present case, after the occurrence of bullae, even with sensitive antibiotic treatment and surgical intervention, the patient died. The patient may have died owing to not only delayed diagnosis and treatment but also the patient’s medical condition.

In conclusion, *V. vulnificus* is a deadly and opportunistic human pathogen that usually infects humans through seafood consumption or direct contact with open wounds. Infection with this pathogen can rapidly progress to septic shock and even death. Therefore, early culture and diagnosis are crucial. Our case alerts clinicians to a possible *V. vulnificus* infection in patients with an unclear infection status, who experience pain of unknown origin in the lower legs, especially if the patient has hypotensive shock, leukopenia, hypoalbuminemia, coagulation dysfunction, increased creatine kinase, or underlying liver disease and diabetes.

## Data Availability

The data that support the findings of this study are available from the corresponding author (Yong Gao).

## Abbreviation

*V. vulnificus*: *Vibrio vulnificus*
